# Mechanisms of persistent atrial fibrillation and recurrences within 12 months post-ablation: Non-invasive mapping with electrocardiographic imaging

**DOI:** 10.3389/fcvm.2022.1052195

**Published:** 2022-11-28

**Authors:** Ramya Vijayakumar, Mitchell N. Faddis, Phillip S. Cuculich, Yoram Rudy

**Affiliations:** ^1^Department of Surgery, Washington University School of Medicine, St. Louis, MO, United States; ^2^Cardiac Bioelectricity and Arrhythmia Center, Washington University in St. Louis, St. Louis, MO, United States; ^3^Division of Cardiology, Department of Medicine, Washington University School of Medicine, Barnes-Jewish Hospital, St. Louis, MO, United States; ^4^Department of Biomedical Engineering, Washington University in St. Louis, St. Louis, MO, United States

**Keywords:** panoramic mapping, human persistent atrial fibrillation, catheter ablation, postablation arrhythmia recurrence, electrocardiographic imaging (ECGI)

## Abstract

**Introduction:**

Catheter ablation of persistent AF has not been consistently successful in terminating AF or preventing arrhythmia recurrences. Non-invasive Electrocardiographic Imaging (ECGI) can help to understand recurrences by mapping the mechanisms of pre-ablation AF and comparing them with the patterns of recurrent arrhythmias in the same patient.

**Methods:**

Seventeen persistent AF patients underwent ECGI before their first catheter ablation. Time-domain activation maps and phase progression maps were obtained on the bi-atrial epicardium. Location of arrhythmogenic drivers were annotated on the bi-atrial anatomy. Activation and phase movies were examined to understand the wavefront dynamics during AF. Eight patients recurred within 12 months of ablation and underwent a follow-up ECGI. Driver locations and movies were compared for pre- and post-ablation AF.

**Results:**

A total of 243 focal drivers were mapped during pre-ablation AF. 62% of the drivers were mapped in the left atrium (LA). The pulmonary vein region harbored most of the drivers (43%). 35% of the drivers were mapped in the right atrium (RA). 59% (10/17) and 53% (9/17) of patients had repetitive sources in the left pulmonary veins (LPV) and left atrial appendage (LAA), and the lower half of RA, respectively. All patients had focal drivers. 29% (5/17) of patients had macro-reentry waves. 24% (4/17) of patients had rotors. Activation patterns during persistent AF varied from single macro-reentry to complex activity with multiple simultaneous wavefronts in both atria, resulting in frequent wave collisions. A total of 76 focal driver activities were mapped in 7/8 patients during recurrence. 59% of the post-ablation AF drivers were mapped in the LA. The pulmonary vein region harbored 50% of total drivers. 39% of sources were mapped in the RA. AF complexity remained similar post-ablation. 58% (44/76) of pre-ablation sources persisted during recurrence. 38% (3/8) of patients had macro-reentry and one patient had rotors.

**Conclusion:**

ECGI provides patient-specific information on mechanisms of persistent AF and recurrent arrhythmia. More than half pre-ablation sources repeated during post-ablation recurrence. This study provides direct evidence for drivers that persist days and months after the ablation procedure. Patient-tailored bi-atrial ablation is needed to successfully target persistent AF and prevent recurrence. ECGI can potentially predict recurrence and assist in choice of therapy.

## Introduction

Atrial fibrillation (AF) is the most common clinical cardiac arrhythmia; it is associated with an increased incidence of heart failure and stroke. The mechanisms of AF origination and perpetuation remain highly debated (1–5) due to incomplete understanding of its pathophysiology.

Catheter ablation for electric isolation of pulmonary veins (PV) can effectively control paroxysmal AF and its symptoms (6). However, despite adjustments by adding linear ablation and defragmentation (7), success of catheter ablation in persistent AF has remained substantially lower than in paroxysmal AF, with frequent recurrence of atrial arrhythmias post-ablation.

Non-invasive Electrocardiographic Imaging (ECGI) (8) studies of AF have been published, demonstrating diverse mechanisms (3, 5). Haissaguerre et al. (3) used ECGI to map drivers in persistent AF and targeted them during ablation. Their results showed an acute termination rate of 75% for persistent AF patients. This was further confirmed by results from a multicenter AFACART trial (9). However, they reported significant rate of atrial tachycardia recurrence, requiring further management. Another prospective single-arm study (10) utilized ECGI to guide targeted driver ablation in the cardiac electrophysiology (EP) lab. These studies have established the feasibility of using ECGI for mapping AF drivers. But the studies focused on the clinical endpoint, namely acute AF termination in the EP lab. Therefore, they could not provide meaningful information on the mechanisms of recurrent AF. From a signal processing standpoint, they used phase mapping to identify potential drivers of AF (10). Our previous work (11) has demonstrated that phase maps need to be independently validated by time-domain activation maps in order to rule out false rotors.

To date, mechanisms of recurrent arrhythmias post catheter ablation have not been systematically analyzed with a panoramic bi-atrial mapping tool. The current study aims to fill this gap by mapping persistent AF in patients undergoing *de novo* catheter ablation and comparing the pre-ablation activation to that of post-ablation recurrence in the same patient. The present study utilizes time-domain activation maps as well as phase maps for data analysis.

## Materials and methods

Seventeen subjects underwent ECGI of their persistent AF (continuous AF lasting >7 days) before their first catheter ablation. All patients were in AF at the time of ECGI. Baseline clinical characteristics of these patients are provided in Table 1.

**TABLE 1:** Baseline clinical characteristics.

	Number (%)	Mean (Range)
Age		64 years (55-72 years)
BMI		36 (27-53)
Obesity	14 (82%)	
**Gender**		
Male	12 (71%)	
Female	5 (29%)	
**Race**		
Caucasian	17 (100%)	
Years since diagnosis of AF		6 years (5 months to 30 years)
**Clinical History**		
Heart Failure	3 (18%)	
Hypertension	13 (76%)	
Diabetes mellitus	4 (24%)	
Obstructive sleep apnea	11 (65%)	
Hyperlipidemia	4 (24%)	
Left atrial dimension		>5 cm
Valve disease	6 (35%)	
Average number of Previous DC cardioversion		2
Number of AADs used before AF ablation		2
Patients on amiodarone before AF ablation	5 (29%)	

Patients were referred from Washington University electrophysiology services. All protocols were approved by the institutional review board at Washington University, and informed consent was obtained from all patients.

The ECGI study procedure and signal processing were described in our previous publications (5, 8, 12–15). Our ECGI methodology has been validated extensively in both atria and ventricles (14). Body surface potentials were recorded on 256 locations using a custom hardware and acquisition software by BioSemi (Netherlands). Patients underwent a thoracic computed tomography (CT) scan to obtain patient-specific heart-torso geometry. Electrode localization and careful manual meshing of the atrial epicardium and body surface were performed using Amira. Pre-processing of body surface signals was done as follows: after eliminating bad signals, a first-order high pass Butterworth filter with a cutoff frequency of 0.5 Hz was applied to remove baseline DC. This was followed by a second-order low pass Butterworth filter with a cutoff frequency of 12 Hz to remove high frequency noise. Fibrillatory windows with duration greater than 700 ms were selected for analysis. We used our own custom software, developed and extensively validated in our laboratory, for reconstructing the epicardial potentials and electrograms. We applied an improved phase-mapping algorithm (11) and wavelet-based activation mapping (5), tailored for AF signal analysis. The wavelet-based activation mapping does not require determination of a specific activation time for a complex AF electrogram, thereby avoiding a challenging error-prone procedure. The accuracy of activation time computation at each location was further improved by analyzing it in the context of its neighbors.

The patients underwent radiofrequency (RF) ablation, with a minimum of bilateral antral PV isolation and additional ablation at the discretion of the operators; details are provided in Table 2. Electroanatomic mapping was performed using CARTO (Biosense-Webster). Four patients underwent rotor mapping (RhythmView™, Topera Medical, Lexington, Massachusetts).

**TABLE 2:** Ablation details.

Patient ID	Sex	Age	AF Duration (months)	Ablation details	Recurrence within 12 months post-ablation
1	M	61	180	PVI, roof line, mitral annular (MA) line, inside CS	9 months
2	F	66		PVI, roof line, MA line	6 months
3	F	69	24	wide antral circumferential ablation (WACA), roof line, MA line, inside CS	5 months
4	M	65	12	WACA, roof line, MA line, focal ablation on LA side of interatrial septum, LA floor overlying CS, inside CS and in RA from SVC to Bachman’s bundle	1 day
5	F	66	5	WACA, roof line, MA line, inside CS	None
6	M	61	96	WACA, roof line, MA line, inside CS	1 day
7	F	64	360	rotor ablation and PVI	None
8	M	56	12	WACA, roof line, MA line, inside CS	None
9	M	64	15	WACA, roof line, MA line, CTI	1 day
10	M	65	48	WACA, roof line, mitral annular line, inside CS	1 month
11	M	55	39	Rotor ablation and PVI	None
12	F	69	24	WACA, roof line, MA line, CTI line	1 month
13	M	62	60	Rotor ablation and PVI	2 weeks
14	M	68	60	WACA, roof line, MA line, inside CS	1 month
15	M	68	48	Rotor ablation and PVI	6 months
16	M	72	120	WACA, roof line, MA line, inside CS	None
17	M	58	36	WACA, roof line, MA line, inside CS	Not known

ECGI results were not available to the operators at the time of the procedure. After the ablation, the subjects were followed-up until a first recurrence for up to 12 months. The primary endpoint was the clinical recurrence of atrial tachyarrhythmia, in the form of AF or atrial tachycardia (AT), lasting for at least 30 s. Early recurrences were defined as arrhythmias occurring within 3 months of ablation and late recurrences as those occurring later than 3 months post-ablation (16).

Of the seventeen patients, eleven recurred within 12 months. Eight of them underwent follow-up ECGI study. The follow-up ECGI procedure was the same as pre-ablation, except that pre-ablation computed tomography (CT) images were utilized for heart-torso geometry, to avoid a repeat CT scan. Time-domain activation maps and phase progression maps were computed during pre-ablation and post-ablation AF in the same patient. Activation sources were classified as follows:

Focal Source: Radial activation spread from a point/region on the atrial epicardium.

Rotor: High curvature wavefront which pivots about a center (fixed or meandering) and completes at least one full rotation. The rotors were independently validated using corresponding time-domain activation maps.

Global activation patterns were distinguished between planar wavefronts and wavefronts forming a macro-reentry circuit.

## Results

### Electrocardiographic imaging of pre-ablation atrial fibrillation

A total of 137 fibrillatory windows (71 seconds of AF) were analyzed for all 17 patients. Activation wavefronts exhibited spatial as well as temporal dynamicity during the mapped intervals. Driver activities consisted of single and repetitive focal sources, rotors, macro-reentry circuits, and simultaneous multiple wavefronts undergoing wave breaks and collision on the bi-atrial surface.

A total of 243 focal activities were mapped. All patients had focal drivers. The computed tomography-based bi-atrial geometry was divided into 7 regions to annotate the location of drivers for each patient. 1 indicates the left pulmonary veins and left appendage; 2, right pulmonary veins and posterior interatrial groove; 3, inferior and posterior left atrium; 4, upper half of right atrium and appendage; 5, lower half of right atrium; 6, anterior left atrium and roof; and 7, anterior interatrial groove (3).

**FIGURE 1:**
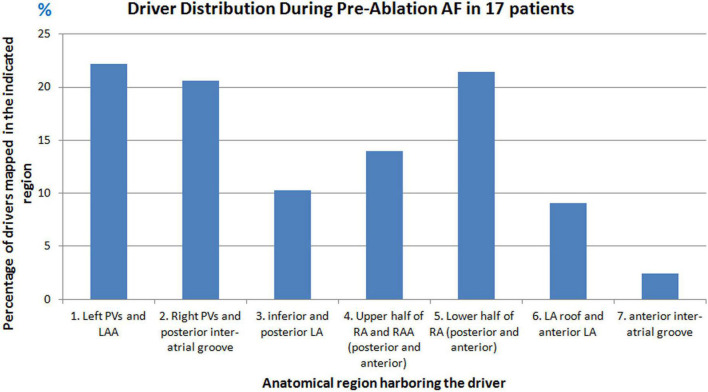
Driver distribution during pre-ablation persistent AF. Graph shows the percentage of drivers mapped from different anatomical regions of the atria in seventeen patients during pre-ablation persistent AF.

Figure 1 shows the percentage of focal drivers mapped for all patients and the corresponding anatomical region from where they were mapped. 62% of the drivers were mapped in the LA, with the pulmonary vein region harboring the most (43%) drivers. 35% of the drivers were mapped in the RA with the lower half of RA harboring 21%.

**FIGURE 2:**
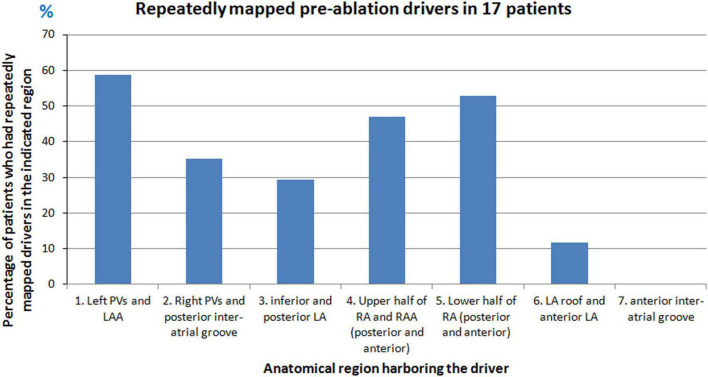
Repetitive sources mapped during pre-ablation persistent AF. Graph shows the percentage of patients who had repetitive drivers from different anatomical regions of the atria during pre-ablation AF.

Repetitive focal sources were mapped from different regions of the atria. A source was considered repetitive if it originated from the same location at least two times either during the same mapping interval or repeated during different mapping intervals. Figure 2 shows the percentage of patients who had repetitive drivers from a particular anatomical region. Overall, the left PV and LAA region harbored frequent drivers in 59% (10/17) of patients. On the RA, the lower half of RA harbored frequent ectopy in 53% (9/17) of patients.

29% (5/17) of patients had macro-reentry wavefronts. 24% (4/17) of patients had rotors. The rotors were mapped in posterolateral RA, LA roof and posterior LA.

### Complexity of pre-ablation persistent atrial fibrillation

Activation patterns during persistent AF varied from single macro-reentry, rotors to complex patterns involving simultaneous wavefronts in the bi-atrial structure, resulting in frequent collisions. On average, two simultaneous wavefronts propagated across the atria.

**FIGURE 3:**
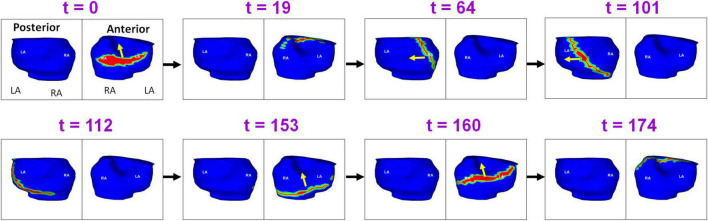
Macro-reentry in persistent AF. Propagating wavefront is shown in red-green. Yellow arrow indicates its direction of propagation. Each rectangular box represents a time instance of bi-atrial activation shown in two views (posterior and anterior). Black arrows between the boxes indicate the chronological sequence of activation. The figure shows activity for 174 ms. LA, Left Atrium; RA, Right Atrium; t, time in milliseconds (Supplementary Video 1).

Figure 3 shows the pre-ablation AF activation pattern due to a single macro-reentry wavefront. A 56-year-old male with 12 months of persistent AF underwent ECGI mapping before his ablation. A fibrillatory window of 780 ms duration was analyzed. The figure shows activity during 174 ms. Propagating wavefront is shown in red-green. Yellow arrow indicates its direction of propagation. Each rectangular box represents a time instance of bi-atrial activation shown in two views (posterior and anterior). Black arrows between the boxes indicate the chronological sequence of activation. Activation originates as single wavefront encompassing the anterior right and left atria (*t* = 0; *t* = 19). The wavefront propagates superiorly, encompassing the posterior right and left atria (*t* = 64; *t* = 101; *t* = 112). It then travels across the floor back to the location of its origin (*t* = 153; *t* = 160). This pattern repeated during the mapped interval. Supplementary Video 1 shows the corresponding movie.

**FIGURE 4:**
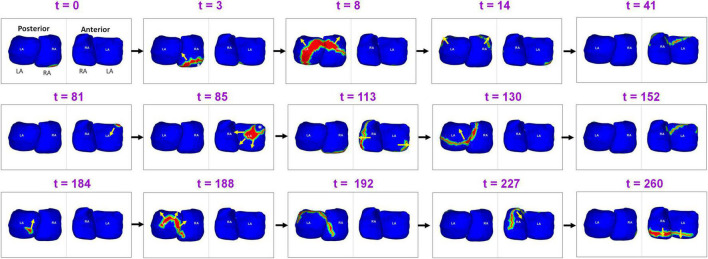
Ectopic sources in left and right atrium and the resulting global activation pattern of a single wavefront during pre-ablation persistent AF. The figure shows activity for 260 ms (Supplementary Video 2).

Figure 4 shows the presence of ectopic sources in both left and right atrium during persistent AF, and the resulting global activation pattern of a single wavefront. A 55-year-old male with 39 months of persistent AF underwent ECGI mapping before his ablation. A fibrillatory window of 834 ms duration was analyzed. The figure shows activity during 260 ms. Activation originates from a focal source on the posterior RA floor (*t* = 0; *t* = 3). The wavefront propagates superiorly, encompassing the posterior right and left atria (*t* = 8; *t* = 14). This wavefront terminates at the roof (*t* = 41). A second wavefront originates from the anterior LA roof near the left atrial appendage (LAA; t = 81) and propagates anteriorly, encompassing both atria. The wavefront returns to the roof at *t* = 152. A third wavefront originates distal to the right inferior pulmonary vein (RIPV; *t* = 184) and travels toward the posterior roof. The wavefront then propagates across the anterior atria and ends at the anterior atrial floor. Supplementary Video 2 shows the entire activation sequence for 834 ms.

**FIGURE 5:**
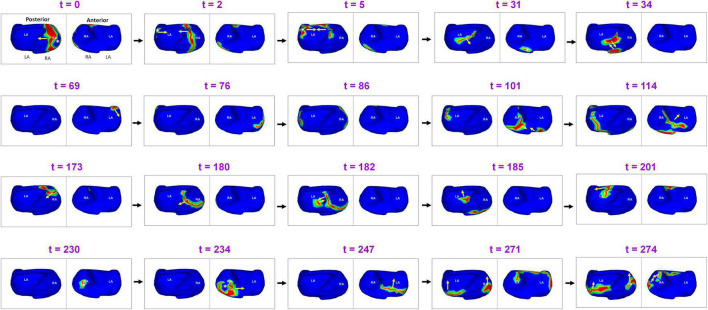
Complex wavefront activation due to multiple simultaneous wavefronts during pre-ablation persistent AF. The figure shows activity for 274 ms (Supplementary Video 3).

Figure 5 shows complex wavefront activation due to multiple simultaneous wavefronts during pre-ablation AF. A 66-year-old female with persistent AF underwent ECGI mapping before her ablation. A fibrillatory window of 370 ms samples duration was analyzed. A wavefront originates in the posterior RA and a branch of the wavefront propagates on the posterior RA parallel to the crista terminalis. At *t* = 2, another wavefront originates from a source in the LSPV-LAA region and travels along the LA roof. It subsequently collides with the previous wavefront at the posterior LA roof. This collision causes a change in direction of activation. The resulting wavefront travels inferiorly along the posterior septum toward the posterior septal floor. At *t* = 31, activity originating from the tricuspid annulus generates a wavefront that propagates toward the posterior septal floor. It collides with the wave traveling along the posterior septum (*t* = 34). At *t* = 69, a wavefront originating from the anterior LAA propagates along the LA free wall. The anterior wavefront collides with another wavefront propagating along the RA lateral wall at the anterior septal floor (*t* = 101), causing it to change direction (*t* = 114). At *t* = 173, source from the anterior septal roof generates a wavefront that propagates toward the posterior RA. It then collides with activity from a source distal to RIPV at the mid posterior septal region (*t* = 182). This collision causes a change in direction of activation. At *t* = 230, a wave originates from a source at the anterolateral RA and branches into two wavefronts which meet again at the RAA at *t* = 274. Supplementary Video 3 shows the full movie for the mapped interval.

**FIGURE 6:**
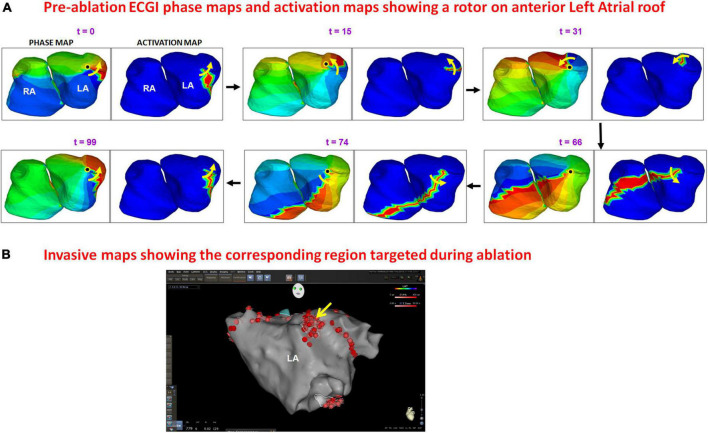
Panel **(A)** shows the phase progression maps (left) and corresponding time-domain activation maps (right) for 100 ms of pre-ablation AF. Phase maps show a counter-clockwise rotor (yellow arrow) on the anterior left atrial roof near the left atrial appendage, with its center of rotation (black dot in phase map) anchored on the roof. The activation maps show high-curvature wavefront completing one full rotation around the same region. The patient underwent rotor ablation and PVI. Panel **(B)** shows the electro-anatomic map of anterior LA obtained during the patient’s ablation. The LA roof region was identified to harbor rotational activity [yellow arrow in panel **(B)**] and was ablated (clustered red dots) by the cardiologist who was blinded to the ECGI finding. The red dots in other regions indicate PV isolation (Supplementary Video 4).

Figure 6 shows an example of rotational activity mapped in a 62-year-old male with 60 months of persistent AF before his ablation. Panel A shows the phase progression maps (left frame at each time t) and corresponding time-domain activation maps (right frame at each time t) for a duration of 200 ms. Phase maps show a counter-clockwise rotor (yellow arrow) on the anterior left atrial roof near the left atrial appendage, with its center of rotation (black dot in phase map) anchored on the roof. The activation maps show a high-curvature wavefront completing one full rotation around the same center. The patient underwent rotor ablation and PV isolation. Panel B shows the electro-anatomic map of anterior LA, obtained during the patient’s ablation procedure. The LA roof region was identified to harbor a rotational activity (yellow arrow in panel B). This was ablated (clustered red dots) by the cardiologist who was blinded to the ECGI finding. Supplementary Video 4 shows the corresponding movie.

### Electrocardiographic imaging analysis of recurrent atrial fibrillation

Eleven patients had post-ablation recurrent atrial arrhythmias within the 12 month follow-up period. Eight of them were available for a follow-up study. ECGI analysis was performed on a total of 64 fibrillatory windows (35 seconds) for the eight patients during recurrent AF. This was compared to activity in 67 fibrillatory windows (35 seconds) of pre-ablation AF for the same patients. A total of 76 focal sources and the corresponding generated activation were mapped during recurrence. 88% (7/8) of patients had focal drivers during recurrent AF. 38% (3/8) of patients had macro-reentry and one patient had rotors.

**FIGURE 7:**
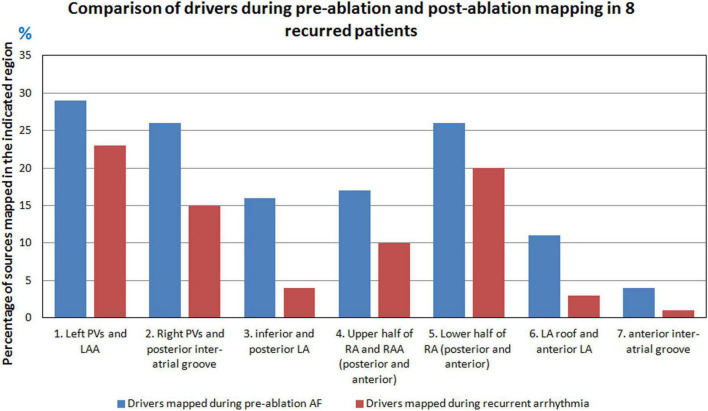
Comparison of pre- and post-ablation AF drivers in the eight patients with recurrent AF.

Figure 7 shows the comparison of pre-ablation and post-ablation drivers for the eight patients and the corresponding anatomical region from where they were mapped. 59% of the post-ablation AF drivers were mapped in the LA, with the pulmonary vein region harboring 50% of total drivers. 30% of sources were from the left PV and LAA region and 20% from right PV and posterior inter-atrial groove. 39% of the post-ablation AF drivers were mapped in the RA with the lower half of RA harboring 26% of sources.

**FIGURE 8:**
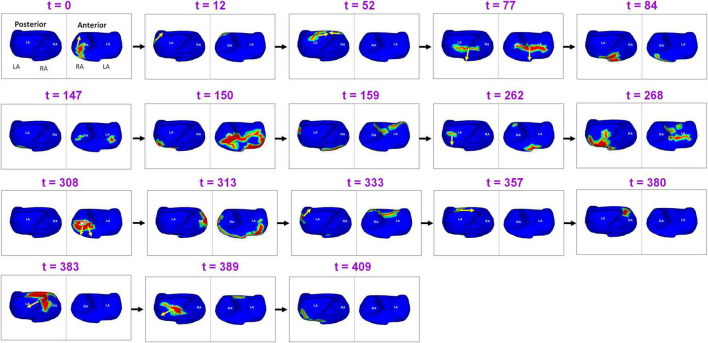
Recurrent AF maps of a patient, obtained 6 months after ablation. The maps show the presence of pulmonary and non-pulmonary sources and the resulting wavefront dynamics (Supplementary Video 5).

Figure 8 shows the complexity of post-ablation AF activation. The example shows that AF is perpetuated by both pulmonary and non-pulmonary sources with wavefront collisions. AF maps were recorded during recurrence, 6 months after ablation. Activation starts near the RA lateral wall floor and the wavefront propagates superiorly along the RA lateral wall. At *t* = 12, another wavefront from a source on the LA posterior wall near the LSPV travels along the LA roof. At *t* = 52 it merges with the wavefront from the RA on the septal roof. After the collision, the two branches propagate toward the septal floor both anteriorly and posteriorly and merge at the septal floor. At t = 147, activation originates across the left and right annular region and proceeds superiorly and inferiorly to join at the roof (*t* = 159). At *t* = 262, activation originates near LIPV, RAA and anterior interatrial septal floor. Wave collisions occur at the posterior septal floor and anterior LA roof. At *t* = 308, a focal source occurs on the RA lateral wall and the resulting wavefront encompasses the anterior atria with one branch traveling along the LA roof (*t* = 313; *t* = 333). This source was also mapped during pre-ablation AF. At *t* = 380, activation originates on the posterior RA roof near the septum. The wavefront branches toward the posterior LA and anterior RA roof (*t* = 383). The branch along the posterior LA roof collides with the previous wavefront (*t* = 383). The resulting wavefront propagates across the posterior LA toward the floor (*t* = 409). Supplementary Video 5 shows the corresponding movie.

**FIGURE 9:**
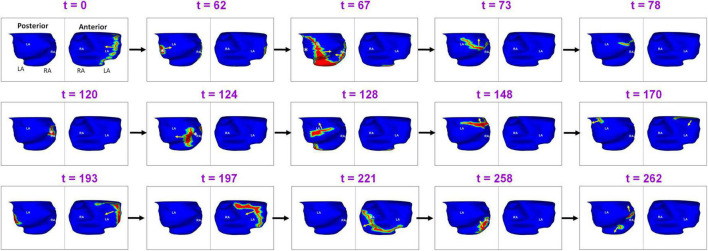
Another example demonstrating the complexity of post-ablation recurrent AF (Supplementary Video 6).

Another example demonstrating the complexity of recurrent AF is shown in Figure 9. A 64-year-old male with 15 months of persistent AF underwent ECGI mapping upon recurrence a day after ablation. Activation map was obtained for a fibrillatory window of duration 560 ms. The activation wavefront propagates from the LA to the RA anteriorly, and then across the posterior RA. A focal source (white asterisk) occurs at the posterolateral LA (*t* = 62). The activation from this source merges with the previous wavefront coming from posterior RA at the posterior septal floor (*t* = 67). This source was also mapped during pre-ablation AF. The resulting wavefront travels superiorly toward the RIPV-mid posterior septum (*t* = 73; *t* = 78). At *t* = 120, a focal source occurs at the RIPV region and the resulting wavefront branches into two. The left branch of the wavefront breaks further. One of its branches activates the posterior LA superiorly while the other branch travels toward the floor (*t* = 128). The superior branch merges with the wavefront activating the posterior RA at the roof (*t* = 148). The resulting wavefront propagates along the LA roof toward the LAA (*t* = 170). At *t* = 193 and *t* = 197, this wavefront propagates from left to right anteriorly (similar to time *t* = 0) and then across the anterior RA (*t* = 221) and posterior RA (*t* = 258). At the mid posterior septal region, the posterior wave breaks and one branch propagates superiorly toward the roof and the other toward the floor (*t* = 262). Supplementary Video 6 shows the corresponding movie.

### Persistence of pre-ablation sources during recurrent atrial fibrillation

**FIGURE 10:**
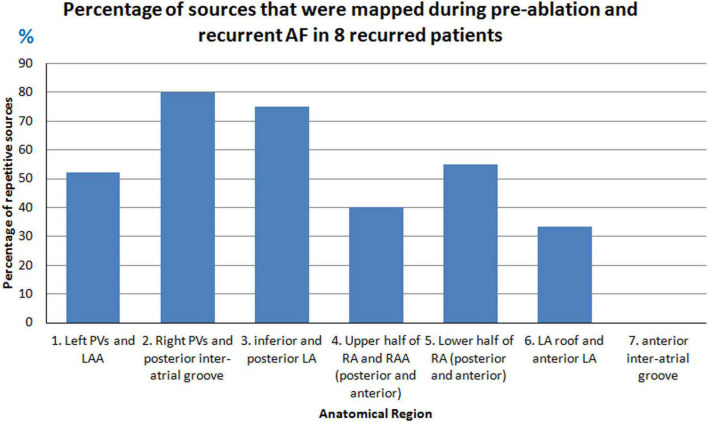
Percentage of pre-ablation sources that persisted during post-ablation recurrence.

A total of 58% (44/76) of sources were mapped during both pre-ablation and recurrence. Figure 10 shows the percentage of sources that were mapped during pre-ablation AF and persisted during recurrence. Overall, the Right PVs and posterior inter-atrial groove regions had the greatest percentage of drivers that persisted after ablation.

**FIGURE 11:**
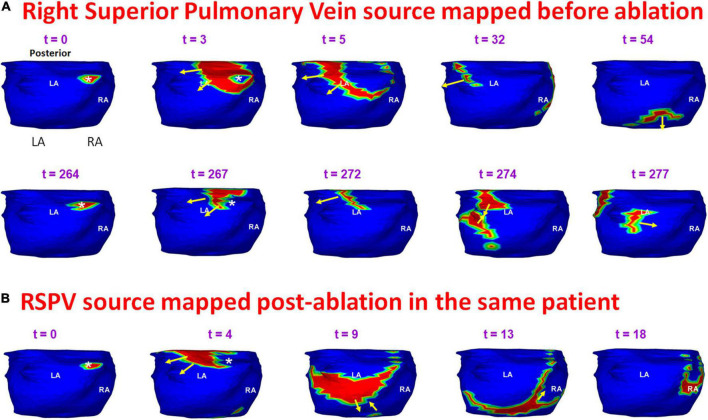
Persistence of pre-ablation RSPV source during recurrent AF. Panel **(A)** shows pre-ablation AF driver from the right superior pulmonary vein (white asterisk) mapped twice (*t* = 0, *t* = 264 ms) within the same mapping interval. This source was repeatedly mapped in this patient pre-ablation. The patient recurred the day after his ablation. Panel **(B)** shows activation maps reconstructed during recurrent AF. The source originates from the same location as pre-ablation ECGI. RSPV – right superior pulmonary vein. Supplementary Video 7 shows the activation during pre-ablation and recurrent AF.

Figure 11 shows an example of a source that was mapped during pre-ablation AF and persisted during recurrence. A 65-year-old male with 12 months of persistent atrial fibrillation was mapped before his first ablation. Panel A shows a pre-ablation AF driver from the right superior pulmonary vein (RSPV; white asterisk) mapped twice (*t* = 0, *t* = 264) within the same mapping interval. A wavefront originates from the RSPV and branches at the posterior septal roof (*t* = 3; *t* = 5). Both branches propagate along the roof toward the LA free wall and RA lateral wall, respectively (*t* = 32). The branches merge at the posterior septal floor (*t* = 54). A second wavefront originates from the same location (*t* = 264). This time it collides with another wavefront approaching from the LA lateral wall (*t* = 274) near the LAA-LSPV region. The collision changes the direction of wavefront propagation (*t* = 277). The RSPV source and associated pattern of activation were repeatedly mapped in this patient pre-ablation. The patient recurred the day after his ablation. Panel B shows activation maps reconstructed during recurrent AF a day after ablation. The post-ablation recurrent source originates from the same location as pre-ablation. The wavefront propagates from the roof toward the floor, where it collides with another wavefront arriving from the septal floor (*t* = 9; *t* = 13). This collision causes a change in wavefront direction. The resulting wavefront propagates toward the posterior right atrial roof. Supplementary Video 7 shows the activation during pre-ablation and post-ablation recurrent AF.

**FIGURE 12:**
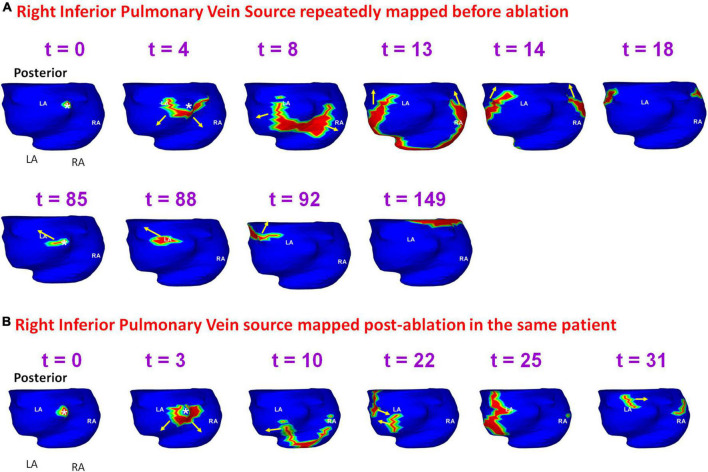
Persistence of pre-ablation RIPV source during recurrent AF. Panel **(A)** shows the activation originating at the RIPV (*t* = 0, *t* = 85 ms) within the same mapping interval during pre-ablation AF. The RIPV source was mapped again during recurrent AF as shown in panel **(B)**. RIPV – right interior pulmonary vein. Supplementary Video 8 shows the activation during pre-ablation and recurrent AF.

Figure 12 shows another example of a pre-ablation dominant source that persisted during recurrent AF, mapped in the patient from Figure 8. Panel A shows the activation originating at the RIPV (*t* = 0) and the wavefront spreads radially, encompassing the posterior left and right atria. At *t* = 85, the same RIPV region gets activated again. This time the wavefront propagates superiorly toward the LA roof and then across the septal roof toward RA. The RIPV source was mapped again during recurrent AF. Panel B shows the radial activation pattern from the recurrent RIPV source (*t* = 0; *t* = 3). The wavefront branches at the posterior septal floor. One branch travels superiorly along posterior LA toward the roof, where it collides with another wavefront that originated at the LAA (*t* = 22). The other branch travels along the RA lateral wall toward the RA roof where it meets the wavefront arriving from the LA roof (t = 31). Supplementary Video 8 shows the activation for the entire fibrillatory window mapped during pre-ablation and post-ablation recurrent AF.

**FIGURE 13:**
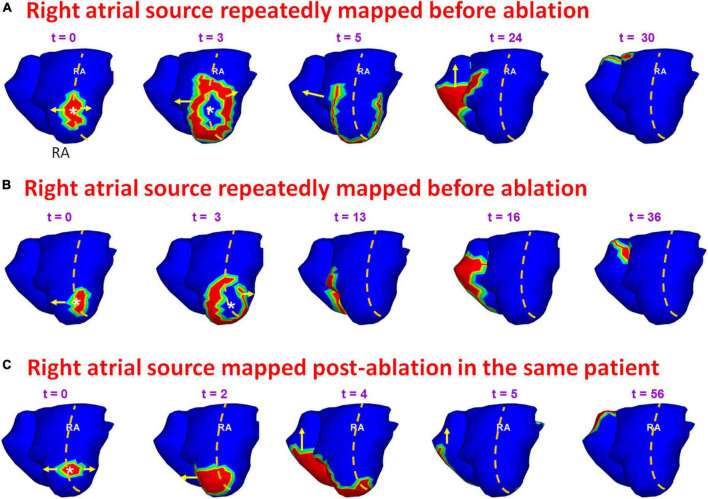
Persistence of pre-ablation RA source during recurrent AF. Panels **(A,B)** show time sequence of wavefront activation originating from a source in posterolateral RA near the floor, reconstructed for two different time intervals during pre-ablation ECGI. Panel **(C)** shows the activation maps reconstructed during post-ablation ECGI. Similar to the pre-ablation activity, a source originates in the posterolateral right atrium near the floor. The activation pattern in panel **(C)** is similar to the pre-ablation wavefront propagation in panels **(A,B)**. Supplementary Video 9 shows the activation during pre-ablation and recurrent AF.

Figure 13 provides another example of a source that was mapped during pre- and post-ablation AF. A 69-year-old female with highly symptomatic persistent AF for 24 months underwent ECGI prior to her first catheter ablation. Panels A and B show time sequence of wavefront activation reconstructed during pre-ablation ECGI for two different time intervals. The crista terminalis is indicated by the orange dotted line along the posterolateral RA. A focal source (white asterisk) originates at the posterolateral RA near the floor (*t* = 0; *t* = 3). The wavefront proceeds parallel to the crista terminalis (*t* = 5 in A; *t* = 13 in B). The wavefront propagates posteriorly across the septum and activates the posterior LA. This source was mapped five times in this patient pre-ablation. The patient recurred within one month of her ablation. ECGI was performed at the 4-month visit. Panel C shows the activation maps reconstructed during post-ablation ECGI. Similar to the pre-ablation activity, a wavefront originates in the posterolateral RA near the floor. The activation pattern in panel C is similar to the pre-ablation wavefront propagation in panels A and B. Supplementary Video 9 shows the activation for the entire fibrillatory window mapped during pre-ablation and post-ablation recurrent AF.

## Discussion

The present study employed non-invasive panoramic mapping to study pre- and post-ablation mechanisms of persistent AF in the same patient. The results demonstrate that persistent AF is perpetuated by macro-reentry wavefronts, rotors and single and repetitive LA and RA focal sources that lead to simultaneous multiple wavefronts propagating in the bi-atrial structure. The wavefronts undergo frequent wave breaks and collisions. These findings concur with our previous study (5) as well as other studies (3). The frequent wave breaks are likely due to structural remodeling of the atria in persistent AF (16). The pulmonary veins region harbored 43% of the total pre-ablation drivers. RA harbored 35% of drivers.

Pre-ablation ECGI analysis also showed that certain anatomical regions harbor repetitive ectopic activity. Most repeated driver activity was mapped in the left PV-LAA region on the LA. This is consistent with the results from Haissaguerre et al., who found that 58% of their 103 patients had repetitive focal sources in the left PV-LAA region (3). Interestingly, in our study, the lower RA region had more repetitive drivers than the upper RA due to the presence of frequent ectopy on the posterior RA floor, crista terminalis and the peri-annular region.

Zhao et al. (17) have characterized AF structural arrhythmic substrates by combining high−resolution optical mapping with 3D structural analysis of wall thickness, fibrosis distribution, and myofiber architecture of explanted human atria. Their results show that structural remodeling of the atria plays a key role in sustaining AF. The locations of sources determined non-invasively using ECGI were consistent with the AF driver regions (superior and inferior regions of posterior LA, and lateral RA close to the crista terminalis) identified in their work.

Being a non-invasive mapping tool, ECGI was successfully used for studying recurrent AF outside the EP lab in 8 patients. The complexity of post-ablation recurrent AF is comparable to that of pre-ablation AF. The results show that recurrent AF is maintained by PV sources associated with pulmonary vein reconnection, as well as by persistence of LA and RA sources mapped before ablation. 50% of the sources mapped post-ablation were from the pulmonary veins region. This suggests pulmonary vein reconnection to be one of the main mechanisms of recurrence.

Another important finding from the study is that 56% of sources mapped pre-ablation also persisted during recurrent AF. This provides direct evidence for drivers that persist days and months after the ablation procedure. The post-ablation recurrent sources were also repeatedly mapped during pre-ablation AF. A prospective study showed that targeting repetitive pre-ablation focal drivers was more likely to result in AF termination (9, 10). Together, these findings emphasize the need to incorporate target-based ablation techniques for increasing the success of AF termination in persistent AF patients.

11 of 17 patients who recurred had pre-ablation repetitive sources, mostly in inferior and posterior LA. This suggests that pre-ablation repetition of sources in these regions can be a predictor of post-ablation recurrence. If established through a larger study, non-invasive ECGI can be potentially used for predicting arrhythmic recurrence in patients considered for catheter ablation. This information could assist in the choice of therapy between catheter ablation, surgery (maze procedure) and drugs.

In our study, we mapped many more focal drivers compared to rotors during both pre-ablation and post-ablation recurrent AF. We used an improved phase analysis approach (11) and time-domain activation maps to confirm the rotors found in phase maps. The higher incidence of rotors in other studies (3, 10) could be due to the simpler phase-based signal processing used exclusively for their analysis. Moreover, ablation of a majority of rotational drivers did not demonstrate a clinically desirable ablation response compared to ablation of focal drivers (10). This could further indicate false detection of rotors in their studies.

### Limitations

Electrocardiographic imaging reconstructs focal sources on the epicardial surface of the atria. Some studies have hypothesized that ectopic activity can also result from local micro-reentry circuits that may appear as focal sources (18); ECGI cannot differentiate between the two. In the ventricle, ECGI can differentiate between an epicardial focal source and epicardial breakthrough of an intramural wave based on the local electrogram morphology (pure Q-wave for an epicardial source; rS morphology for breakthrough). This is very challenging for the low voltage complex electrograms of AF. Also, studies performing simultaneous endocardial and epicardial mapping of AF on the left atrial free walls of goats have shown dissociation of activity between the epicardium and endocardium (19); this property of AF cannot be studied with ECGI. Despite these limitations of epicardial-only mapping, ECGI-guided ablation plus PV isolation resulted in high degree of freedom from AF during follow-up and a clinically favorable ablation response in a large proportion of patients (10).

Due to the small number of patients in this study, a statistically meaningful conclusion on correlation between the clinical parameters, hospital stay and recurrences could not be derived.

## Data availability statement

The raw data supporting the conclusions of this article will be made available by the authors, without undue reservation.

## Ethics statement

The studies involving human participants were reviewed and approved by Washington University Institutional Review Board. The patients/participants provided their written informed consent to participate in this study.

## Author contributions

RV conducted all experiments and generated the figures and movies of this manuscript. All authors have interpreted the results, wrote the manuscript, and approved the submitted version.
